# Gut Microbiome-Targeted Nutrition Interventions and Growth among Children in Low- and Middle-Income Countries: A Systematic Review and Meta-Analysis

**DOI:** 10.1016/j.cdnut.2024.102085

**Published:** 2024-02-14

**Authors:** Hammond Yaw Addae, Charles Apprey, Alexander Kwarteng

**Affiliations:** 1 Department of Biochemistry and Biotechnology, Kwame Nkrumah University of Science and Technology (KNUST), Kumasi, Ghana; 2 Nursing Department, Nursing and Midwifery Training College, Kpembe, Ghana; 3 Kumasi Centre for Collaborative Research in Tropical Medicine, KNUST, Kumasi, Ghana

**Keywords:** child gut microbiome, low- and middle-income countries, meta-analysis, microbiome-directed complementary feeds, nutrition intervention studies, probiotics, synbiotics, systematic review

## Abstract

**Background:**

Childhood malnutrition is a public health challenge of much interest and concern globally. However, a perturbed gut microbiome (GM) may limit some nutrition interventions’ effects among healthy children with undernutrition.

**Objectives:**

This review aimed to evaluate the effects of GM-targeted nutrition interventions on growth outcomes among children (0–59 mo) using published studies in low- and middle-income countries.

**Methods:**

The methods were guided by the Cochrane methodology. The literature search was conducted to include articles published from inception to July 2023 in PubMed, Google Scholar, and Cochrane Databases. We identified and included 35 studies among 11,047 children. The analysis was conducted considering various growth parameters in the qualitative synthesis and weight gain (kg) in the meta-analysis.

**Results:**

In the qualitative synthesis, 55.6% of prebiotics, 66.7% of probiotics, 71.4% of synbiotics, and 28.6% of “microbiome complementary feed” studies had significant effects on growth outcomes. Also, prebiotics had more studies with significant effects among healthy children, whereas probiotics, synbiotics, and “microbiome complementary feeds” had more studies with significant effects among children with undernutrition. Nineteen studies were included in the meta-analyses, of which 7 (36.8%) measured GM outcomes. The meta-analysis showed that prebiotics exhibited heterogeneity but had significant effects on weight in the intervention as compared with the control (mean difference [MD]: 0.14 kg; 95% CI: 0.02, 0.25; I^2^ = 63%, *P* = 0.02; 4 studies, *n* = 932). Probiotics had significant effects on weight in the intervention (MD: 0.15 kg; 95% CI: 0.06, 0.25; I^2^ = 42%, *P* = 0.05; 8 studies, *n* = 2437) as compared to the control. However, synbiotics (MD: 0.26 kg; 95% CI: –0.04, 0.56; I^2^ = 41%, *P* = 0.17; 4 studies, *n* = 1896] and “microbiome complementary feed” (MD: –0.03 kg; 95% CI: –0.18, 0.11; I^2^ = 0%, *P* = 0.60; 3 studies, *n* = 733] had no significant effects on weight in the intervention as compared with control.

**Conclusions:**

Although probiotics and synbiotics may be effective at enhancing growth among children, the selection of interventions should be contingent upon health status.

This trial was registered at www.crd.york.ac.uk/prospero/ as CRD42023434109.

## Introduction

Globally, childhood malnutrition is a public health challenge of much interest and concern [1]. It affects an estimated 149 million children aged <5 y, according to the WHO [1], in low-and middle-income countries (LMICs). These countries account for most child deaths from preventable causes, such as diarrhea, pneumonia, and malnutrition [2]. The human gut microbiome (GM) is a complex ecology of microorganisms that coexist with its human host. If unperturbed, such coexistence is usually a mutually beneficial relationship [3]. Such a relationship plays a significant role in human physiological processes, including brain function [4] and immunity [5]. The GM goes through changes throughout the lifecycle particularly from infancy to preschool age. Notable changes, occasioned by the changes in feeding mode, occur during the transition from exclusive breastfeeding to complementary feeding and later during weaning [6]. Other factors, such as the child’s mode of delivery, antibiotics intake, environmental exposures, and geographic location all have influence in shaping GM as a child ages [3]. Children in LMICs are particularly vulnerable to GM perturbations because of poor water and sanitation, inadequate dietary intake, and infectious diseases coupled with recent rising cesarean section rates [7].

Malnutrition is associated with impaired GM development, reduced immune function, and increased susceptibility to infections and chronic diseases [3]. GM dysbiosis which is characteristic of susceptible children or children with undernutrition, may limit the effects of nutrition interventions at promoting growth or enhancing recovery [8]. The imbalance between beneficial and harmful bacteria associated with GM dysbiosis could enhance the overgrowth of specific bacterial species, such as *Clostridium difficile* that could compete for intervention nutrients and hence reduce nutrient availability to the host. Therefore, GM-targeted nutrition interventions have been proposed as a promising strategy to improve child nutritional outcomes. As such, in recent times, nutrition interventions, aside from enhancing growth, have sought to target GM dysbiosis by using prebiotics, probiotics, synbiotics, or specialized complementary feeds [3,9].

Prebiotics are the nondigestible components of carbohydrates such as inulin, galacto-oligosaccharides (GOS), and fructo-oligosaccharides (FOS) that resist breakdown in the small intestine and reach the large intestines intact, where they serve as a food source for the gut microbiota [10]. Among other things, prebiotics stimulate the production of short-chain fatty acids [11]. It also helps improve gut barrier function and delay gastric emptying. To the contrary, probiotics are themselves living microorganisms that, when consumed in appropriate quantities, provide health benefits to the host [10]. Probiotics are typically beneficial bacteria from the Lactobacillus or Bifidobacterium genus that play a significant role in maintaining a healthy digestive system and overall health [10]. They feed on prebiotics and enhance nutrient absorption, improve immune function [5], reduce inflammation, and promote a healthy balance of bacteria in the gut. However, synbiotics are a combination of prebiotics and probiotics that work together to confer health benefits to humans [12]. The synergistic effects of combining probiotics and prebiotics may be achieved when both are administered concurrently [13]. By combining the 2, synbiotics aim to enhance the survival and activity of probiotic bacteria in the gut, in addition to being a food source for their growth.

Although GM is an important factor that modifies the effects of nutrition interventions on a child’s nutritional status, previous reviews on this subject have been among only healthy infants [14,15]. Some other systematic reviews have centered on both LMICs and high-income countries [16] or included interventions among only infants aged <1 y [17]. However, the only reviews examining the effects of prebiotics, probiotics, and synbiotics on childhood growth in the context of LMICs are publications by Onubi et al. [18] and Heuven et al. [8]. Although the former was published about a decade ago, the latter excluded children aged <6 mo. Heuven et al. [8] also excluded interventions with durations <12 wk. Because children of some undernutrition interventions recover before the twelfth week, it is possible that Heuven et al. [8] may have missed publications of some critical malnutrition interventions. Additionally, Heuven et al. [8] did not include studies that used gut “microbiome complementary feeds” as an approach to enhancing growth. Therefore, this systematic review aimed to evaluate the effects of microbiome-directed nutrition interventions (prebiotics, probiotics, synbiotics, and “microbiome complementary feeds”) on growth outcome(s) among children (0–59 mo) using published controlled trials in LMICs. Such a review may inform policy and practice by identifying the most effective and feasible interventions, as well as identifying research gaps and priorities for future studies.

## Methods

This review was registered with the PROSPERO and is available at www.crd.york.ac.uk/prospero/ as CRD42023434109. The methods and procedures used in this systematic review followed the PRISMA checklist [19] and the Cochrane Handbook for Systematic Reviews of Intervention Studies [20].

### Inclusion and exclusion criteria

The review included all types of controlled trials and studies of GM-targeted dietary interventions directed at enhancing child growth in LMICs. The criteria for classifying a country as an LMIC was based on the World Bank classifications [21]. The interventions included any type of “microbiome complementary feeds,” prebiotic, probiotic, or synbiotic, delivered alone or through any vehicle such as milk, infant formula, isotonic solutions, maltodextrin, complementary foods, or any nutrition rehabilitation feed, such as formula-75 (F-75), formula-100 (F-100) or ready-to-use therapeutic feed (RUTF). The term “microbiome complementary feed(s)” is used reservedly to denote all GM-targeted dietary intervention studies that sought to enhance growth but did not specifically mention the use of probiotics, prebiotics, synbiotics, or their derivatives. The outcomes of interest included any measure of GM composition or function, as well as nutrition outcomes, such as growth (weight gain, height-for-age *z*-score, weight-for-height *z*-score, weight-for-age *z*-score, BMI, stunting, wasting or underweight) or blood outcome parameters such as serum ferritin, serum albumin or hemoglobin. For intervention on complementary feeds to be included, they should report both GM and growth outcomes.

This review excluded studies of animal models. Prenatal GM studies among women during pregnancy but with outcomes on infants’ GM and studies of child GM with other outcome measures aside from nutrition, such as irritable bowel syndrome, were all excluded. It also excluded GM studies involving preterm babies and studies with part or all participants living in high-income countries. For synthesis, the interventions were grouped into 4, i.e., prebiotics, probiotics, synbiotics, and “microbiome complementary feed” studies.

### The search strategy

The literature search was conducted in PubMed, Google Scholar, and Cochrane Library electronic databases. This was done to identify intervention studies published from inception till July 2023. The literature search was carried out from June 2023 to July 2023. Grey literature, such as reference lists of relevant reviews and studies, were also included in the search. EndNote software version X7 (Clarivate Analytics) was used to detect and expunge duplicate studies. Two authors (HYA and CA) independently reviewed and screened the titles and abstracts of the identified studies and, subsequently, the full texts of the potentially relevant studies, using predefined exclusion and inclusion criteria. Identified discrepancies were resolved through discussion and consensus between the 2 authors in consultation with the main supervisor. Details of the search strategy have been included and attached as Supplementary File 1.

### Data extraction

The data extraction was performed manually using a prepiloted standardized form that includes study characteristics such as author and year, study country, sample size, age of participants, dietary intervention and duration, method of bio-specimen analysis, health status, delivery vehicle(s), growth outcome(s), GM outcome(s) main objective(s), main finding(s) and whether the intervention had a significant positive effect. This data extraction was carried out by 2 authors independently. The standardized forms were then compared after data extraction, and discrepancies were resolved with the inputs of the main supervisor.

### Outcome variables

Height or length, weight, height-for-age, weight-for-height, weight-for-age, wasting, stunting, underweight, BMI, or blood parameters such as hemoglobin concentrations, albumin, or serum ferritin were the outcomes of interest in this review. They served as the basis for deciding whether an intervention has had the desired effect on participants. Other outcome variables associated with gut health and microbiota, such as microbiota-for-age *z*-score and microbiota alpha and beta-diversity, were also noted. However, the weight gain parameter was included in the meta-analysis section. This is because it was the most prevalent nutrition-related outcome indicator among the included studies.

### Quality assessment

The Cochrane Risk of Bias (ROB) tool for randomized control trials [22] was used in evaluating the quality of the included trial studies. Areas assessed included bias arising from the randomization process, bias because of deviation from intended interventions, bias in the measurement of the outcome, bias because of missing outcome data, and bias in the selection of the reported results. A composite rating based on the above adjudged each of the interventions as either “low risk,” “unclear,” or “high risk” of bias. Details of individual ROB scores for all included studies are attached as Supplementary File 2.

### Data analysis

Given that this review had the objective of assessing the effects of various interventions, the end-line, instead of baseline sample sizes, was used in this systematic review. This is because the end-line numbers reflect the participants who completed the study and, therefore, provide a more accurate estimate of an intervention’s effect, i.e., mean difference (MD). Weight gain was defined as the differences in preintervention weight and postintervention weight in the control group and the intervention group. MD referred to the arithmetic differences between the mean weight gain in the control group and the mean weight gain in the intervention group. Only the weight gain parameter was included in the meta-analysis as an outcome variable. This was done to minimize heterogeneity. Weight gain was also used because it is the most prevalent growth-related outcome reported in the included studies. Weight is also a part of the other composite anthropometric measures such as BMI, weight-for-height *z*-score, and weight-for-age *z*-score. Where end-line sample sizes were different for different outcome measures, sample sizes for nutrition-related outcome variables were used for the qualitative synthesis. For the meta-analysis section, forest plots were used to present results. For studies in which heterogeneity was detected (*P* < 0.05 and I^2^ > 50%), the common effects model analysis was used. For studies with no heterogeneity (*P* ≥ 0.05 and I^2^ ≤ 50%), the random effects model analysis was carried out. The meta-analysis included only articles that reported preintervention and postintervention weight and articles that reported weight gain. All meta-analyses were conducted at a 95% confidence interval (CI) using R software version 4.2.3 (R Core Team).

## Results

**FIGURE 1:**
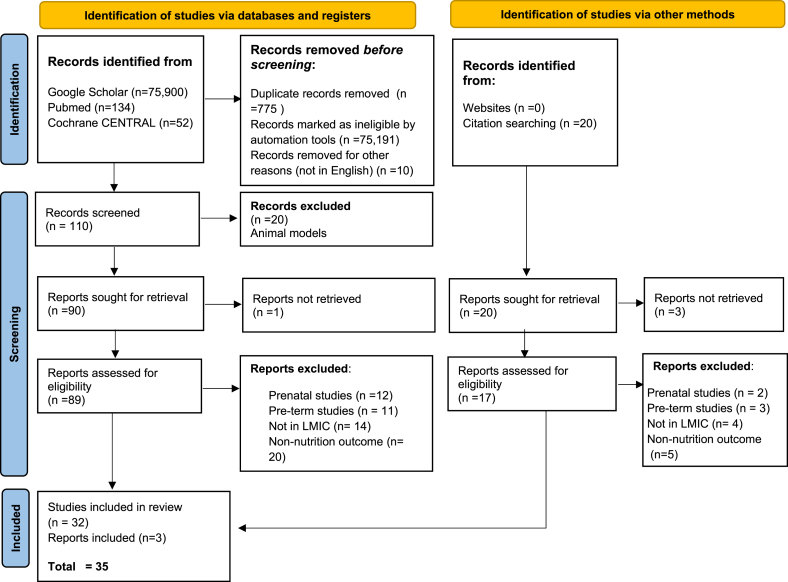
PRISMA 2020 flow diagram for prebiotics, probiotics, synbiotics, and “microbiome complementary feeds” for low- and middle-income countries (LMIC).

The PRISMA flow diagram [19] of the included studies is shown in Figure 1. Overall, 35 studies, all published in the last 2 decades approximately, met the inclusion criteria and were included in this systematic review.

### Summary of all included studies

The number of trials included was 35, and the total number of children analyzed by all studies was 11,047. Geographically, the review included studies from 16 counties, and most of the studies were conducted in Malawi (5, 14.3%) and Indonesia (5, 14.3%). Eleven (31.4%) of the included studies were conducted in Africa, 16 (45.7%) in Asia, 3 (8.6%) in South America and 4 (11.4%) in the Middle East. Twenty (57.1%) studies had a significant effect on ≥1 growth outcome. Also, 14 (40%) studies were among children with undernutrition, whereas 20 (57.1%) studies were among healthy children, and 1 study with a not clearly defined health status. The mean intervention duration was 151.3 d, with a range of 28–360. The summary of the location, intervention duration, sample size, health status, and significant positive effect on growth are shown in Table 1.

**TABLE 1:** Summary of location, health status and effects on growth of all included studies

No.	Author, publication year	Duration (days)^1^	Sample size^2^	Health status	Significant positive effect on growth?	Country	Region
1	Aakko et al., 2017	180	160	Healthy	No	Malawi	Africa
2	Agustina et al., 2013	180	494	Healthy	Yes	Indonesia	Asia
3	Barratt et al., 2022	28	62	Undernourished	Yes	Bangladesh	Asia
4	Batool et al., 2023	56	204	Undernourished	Yes	Pakistan	Middle East
5	Calder et al., 2021	28	58	Undernourished	Yes	Uganda	Africa
6	Chen et al., 2021	90	118	Undernourished	Yes	Bangladesh	Asia
7	Cheung et al., 2016	360	213	Healthy	No	Malawi	Africa
8	Duggan et al., 2003	180	547	Healthy	No	Peru	South America
9	Famouri et al., 2014	180	84	Undernourished	Yes	Iran	Middle East
10	Firmansyah, et al., 2011	365	393	Healthy	Yes	Indonesia	Asia
11	Grenov et al., 2017	84	314	Undernourished	No	Uganda	Africa
12	Hemalatha et al., 2014	270	379	Healthy	No	India	Asia
13	Hughes et al., 2020	360	515	Healthy	No	Malawi	Africa
14	Jones et al., 2015	84	60	Undernourished	No	Kenya	Africa
15	Kamil et al., 2022	50	30	Undernourished	Yes	Indonesia	Asia
16	Kara et al., 2019	90	71	Undernourished	Yes	Turkey	Middle East
17	Kerac et al., 2009	33	795	Undernourished	No	Malawi	Africa
18	Kosuwon et al., 2018	84	119	Healthy	No	Thailand	Asia
19	Kusumo et al., 2019	90	38	Healthy	No	Indonesia	Asia
20	Mai et al., 2020	84	1003	At-risk children	Yes	Vietnam	Asia
21	Nakamura et al., 2006	180	133	Healthy	No	Bangladesh	Asia
22	Nuzhat et al., 2023	28	67	Undernourished	Yes	Bangladesh	Asia
23	Ordiz et al., 2020	168	236	Healthy	No	Malawi	Africa
24	Paganini et al., 2017	120	145	Healthy	Yes	Kenya	Africa
25	Pfluger et al., 2022	180	48	Healthy	Yes	Mali	Africa
26	Rehman et al., 2020	48	30	Undernourished	Yes	Pakistan	Middle East
27	Rehman et al., 2020	48	30	Undernourished	Yes	Pakistan	Asia
28	Ribeiro et al., 2012	108	130	Healthy	No	Brazil	South America
29	Robertson et al., 2023	540	335	Healthy	No	Zimbabwe	Africa
30	Saran et al., 2002	180	100	Undernourished	Yes	India	Asia
31	Sazawal et al., 2010	365	624	Healthy	Yes	India	Asia
32	Silva et al., 2008	101	109	Healthy	Yes	Brazil	South America
33	Sur et al., 2011	84	3229	Healthy	No	India	Asia
34	Surono et al., 2011	90	79	Healthy	Yes	Indonesia	Asia
35	Zambrana et al., 2019	180	95	Healthy	Yes	Mali and Nicaragua	Africa and South America

1 Mean (range) =153.3 (minimum = 28, maximum = 360).

2 Total sample size =11,047.

### Prebiotics

Nine prebiotic studies met the inclusion criteria [[23], [24], [25], [26], [27], [28], [29], [30], [31]], and their study characteristics are presented in Table 2. The interventions were conducted on 1392 children. Six [[25], [26], [27], [28], [29],^31^] of these studies were among healthy children and 3 [23,24,30] among children with undernutrition. Three of the studies used oligosaccharides as the prebiotic, which include GOS [23,24] and FOS [29]. However, 2 of the studies used rice bran [25,27] as the prebiotic. The others used a combination of GOS + iron [26], GOS + polydextrose [28], Oligofructose + Zinc [31], and alpha-linolenic acid from flaxseed as prebiotic [30]. In general, the vehicles for conveying this prebiotics were RUTF among undernourished children and milk or complementary feeds among healthy children. Five [[23], [24], [25], [26], [27]] of the 9 prebiotic trials (55.6%) had significant positive effects on ≥1 growth outcome relative to their respective control groups. Of the 5 that reported significant effects, 3 studies [[25], [26], [27]] were among healthy children and 2 children with undernutrition [23,24]. The mean number of days for intervention duration was 126.2 d, with the least and highest being 48 d and 180 d, respectively.

**TABLE 2:** Characteristics of prebiotic intervention studies

Author, publication year	Study country	Dietary intervention type, duration^1^	Method of bio-specimen analysis	Vehicle(s)	Sample size^2^ (age group)	Health status	Growth outcome(s)	Gut microbiome outcome	Objective(s)	Main finding(s)	Significant positive effect?
1. Batool et al., 2023 [23]	Pakistan	Two-arm study. 4 g/d prebiotic GOS + RUTF or RUTF + 4 g/d starch (placebo) for 8 wk.	Complete blood count analysis	RUTF	204 (6–59 mo)	Undernourished	Weight, MUAC, hemoglobin, hematocrit, platelet, corpuscular volume, albumin	None	To assess the efficacy of prebiotics as a synergistic additive to RUTF to enhance blood parameters and anthropometric measurements in children with uncomplicated SAM.	Supplementation with RUTF and prebiotics has proven to be an efficient, effective, and safe therapy for children suffering from SAM.	Yes
2. Duggan et al., 2003 [31]	Peru	Four-arm study. Only cereal (placebo) or 0.55 g/d OF + cereal only or 1 mg/d zinc + cereal only or 1 mg/d znic + 0.55 g/d OF + cereal for 6 mo.	Blood analysis	Cereal (rice or oat)	547 (6–12 mo)	Healthy	Weight, WAZ, HAZ, plasma zinc	None	To evaluate the effects of dietary supplementation with oligofructose with and without zinc on the prevalence of diarrhea in a community with infections.	Prebiotic supplementation had no effect on the occurrence or severity of gastrointestinal infections or growth.	No
3. Jones et al., 2015 [30]	Kenya	Three-arm study. Standard 92 g/kg/d RUTF, or 92 g/kg/d RUTF containing flax seed oil or 92 g/kg/d RUTF containing flax seed oil + fish oil capsules (containing 214 mg lc PUFA) for 84 d.	Whole blood analysis	RUTF	60 (6–60 mo)	Undernourished	MUAC, WHZ, WAZ, HAZ, head circumference, erythrocyte lc n–3 PUFA	None	To develop a RUTF with elevated lc PUFA and measure its impact, with and without fish oil supplementation, on children’s PUFA status during treatment of SAM.	PUFA requirements and the expected growth of children with SAM are not met by these specialized RUTFs manufactured according to specifications.	No
4. Nakamura et al., 2006 [29]	Bangladesh	Two-arm study. 1 g/d glucose (placebo) or 2 g/d FOS for 6 mo.	Anthropometry only	Isotonic solution	133 (25–59 mo)	Healthy	Weight, height, MUAC	None	To assess the prebiotic FOS effect on body weight and reduction of diarrhea among children	Daily intake of FOS was associated neither with growth nor diarrhea episodes	No
5. Paganini et al., 2017 [26]	Kenya	Three-arm study. MNP only (control) or MNP + 5 mg/d iron or MNP + 5 mg/d iron + 7.5 g/d GOS for 4 mo	16S rDNA sequencing of stool	Maize porridge	145 (6.5–9.5 mo)	Healthy	Hemoglobin, plasma ferritin, c-reactive protein, alpha-glycoprotein	Phylogenetic distance and gut microbiome composition	To evaluate the efficacy and safety of a new MNP formula with prebiotic GOS combined with a low dose (5 mg/d) of highly bioavailable iron	MNP containing a low dose of iron reduces anemia, and the addition of GOS mitigates the adverse effects of iron on the gut microbiome and morbidity.	Yes
6. Pfluger et al., 2022 [25]	Mali	Two-arm study. Rice bran prebiotic at 1 g/d, 2 g/d, 3 g/d, 3 g/d, 4 g/d, 5 g/d for 1st, 2nd, 3rd, 4th, 5th, and 6th mo, and no intervention for 6 mo, respectively.	Nontargeted dried blood spot-based metabolomics	Complementary food	48 (6 mo)	Healthy	Weight, WAZ, HAZ, WHZ and hemoglobin, lipids, and amino acid metabolites	None (methylsuccinate)	To investigate the effects of rice bran supplementation on healthy infant weaning and utilized dried blood spots to identify novel nutrition and metabolic biomarkers via nontargeted metabolite profiling.	These findings support rice bran as a weaning ingredient to meet infant nutritional requirements. This study provides evidence for dried blood spots as a cost-effective tool to detect infant nutritional biomarkers.	Yes
7. Rehman et al., 2020 [24]	Pakistan	Two-arm study. Prebiotic (F-75, 4.1 g/L, and 6.1 g/L GOS; F-100 and RUTF, 5.5 g/L and 8.25 g/L GOS) or placebo for 48 d	Hematologic analysis using venous blood	F-75, F-100, or RUTF	30 (6–59 mo)	Undernourished	Serum albumin, serum glutamic oxaloacetic transaminase, serum glutamic pyruvate, white blood cells, serum electrolyte potassium	None	To elicit the role of prebiotics on the nutritional status of SAM children by measuring some hematologic parameters.	GOS prebiotics supplementation improved blood hematology and decreased the risk of infection among SAM.	Yes
8. Ribeiro et al., 2012 [28]	Brazil	Two-arm study. 0.5 g GOS + 0.5 g PDX per serving or placebo for 108 d	Anthropometry only	Cow’s–milk-based follow-on formula	129 (9–48 mo)	Healthy	WHZ	None	To assess the effects of a formula-supplemented with the prebiotics PDX and GOS on diarrhea and growth in toddlers	The 2 groups had similar weight-for-length/height *z* scores and similar odds of having diarrheal disease.	No
9. Zambrana et al., 2019 [27]	Nicaragua and Mali	Two-arm study. Rice bran prebiotic (age 6–7 m = 1 g/d, 7–8 m = 2 g/d, 8–10 m = 3 g/d, 10–11 m = 4 g/d, 11–12 m = 5 g/d) and no intervention for 6 mo	16S rRNA sequencing of stool	Complementary feed	95 (6–12 mo)	Healthy	WAZ, HAZ, WHZ	Alpha and beta-diversity indices	To investigate the effects of rice bran supplementation on WAZ and length-for-age *z*-score, stool biomarkers, as well as microbiota and metabolome in weaning infants	Rice bran is a practical dietary intervention strategy that merits development in regions that have a high prevalence of growth stunting because of malnutrition.	Yes

F-100, formula-100; F-75, formula-75; FOS, fructo-oligosaccharides; GOS, galacto-oligosaccharides; HAZ, height-for-age *z*-score; lc PUFA, long-chain polyunsaturated fatty acid; MNP, micronutrient powder; MUAC, mid-upper arm circumference; OF, oligofructose; PDX, polydextrose; RUTF, ready-to-use therapeutic feed; SAM, severe acute malnutrition; WAZ, weight-for-age *z*-score; WHZ, weight-for height *z*-score; rDNA, ribosomal Deoxyribonucleic acid.

1 Mean intervention duration (range) = 126.2 d (minimum = 48 d, maximum = 180 d).

2 Total sample size = 1392.

**FIGURE 2:**
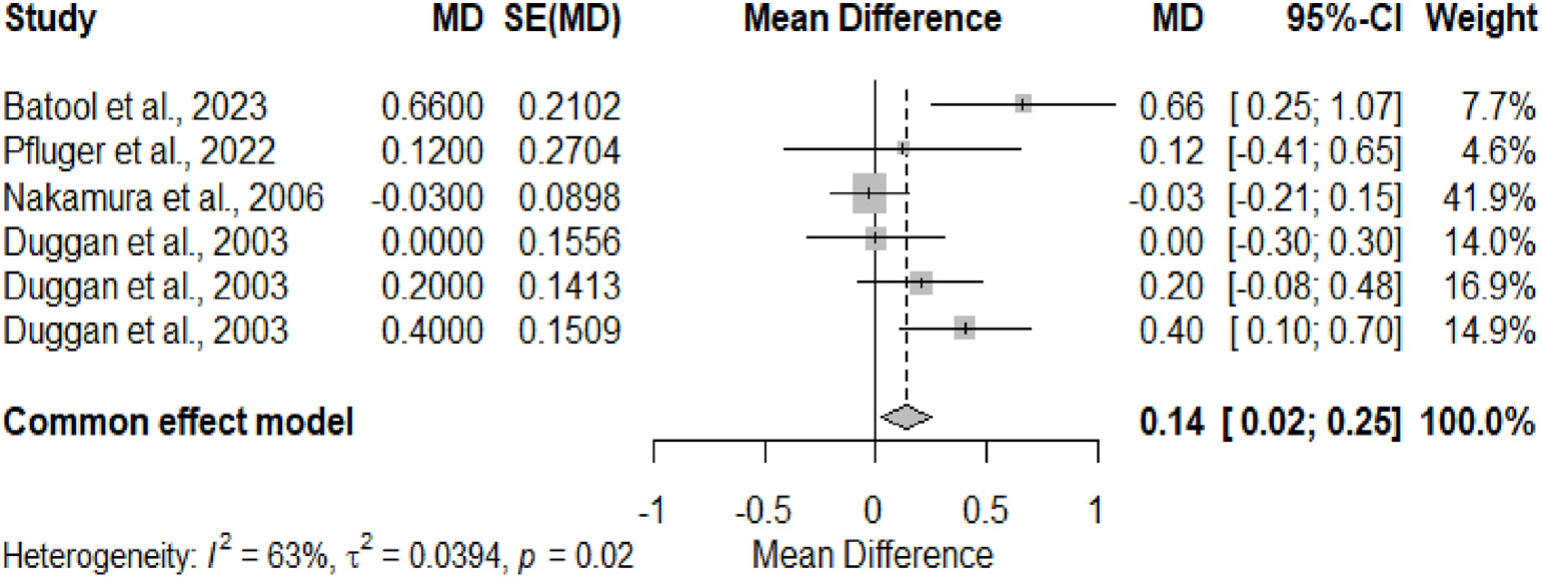
Forest plot of prebiotic studies that reported weight gain. Growth (weight gain) was measured in kg. Weight gain was defined as the differences in preintervention weight and postintervention weight in the control group and the intervention group. MD referred to the arithmetic differences between the mean weight gain in the control group and the mean weight gain in the intervention group. CI, confidence interval; SE, standard error.

Figure 2 shows the heterogeneity descriptions and the effect sizes MD of selected prebiotic studies and growth outcome (weight gain) with a 95% CI. The included prebiotic studies had a significant overall effect on weight gain (MD = 0.14, 95% CI: 0.02, 0.25). However, significant heterogeneity was also identified among the included studies (I = 63%, *P* = 0.02).

### Probiotics

The characteristics of the included studies are outlined in Table 3. The total number of participants analyzed by these studies was 5876 children. Twelve trial studies [[32], [33], [34], [35], [36], [37], [38], [39], [40], [41], [42], [43]] that used probiotics to influence GM and growth met the inclusion criteria. Of these, 5 studies [32,33,35,39,43] were among children with undernutrition, whereas 6 [34,37,38,[40], [41], [42]] were among healthy children, and 1 study [36] was on children whose health status was not sufficiently described (children at-risk). Eight of the studies included the *Lactobacillus* genus only. Specifically, *L. acidophilus* [34,43], *L. plantarum* [35,38], *L. casei* [36,42], *L. rhamnosus* [39] and *L. paracasei* [32]. Three studies used a combination of 2 bacteria species, i.e., *Bifidobacteria lactis* + *L. rhamnosus* [33], *Bifidobacteria lactis* + *L. paracasei* [37], and *L. casei* + *L. reuteri* [40] with the last study [41] using *Enterococcus faecium*. The vehicles of delivery were mainly F-75, F-100, or RUTF for undernourished children and milk or maltodextrin for healthy children. The last and highest duration of intervention was 48 d and 270 d, with the mean duration being 112.6 d, respectively. In all, 8 [32,[34], [35], [36],[39], [40], [41],^43^] out of the 12 included probiotic studies (66.7%) reported a significant positive effect of probiotics on ≥1 growth outcome among the intervention group as compared to the control group. Four [32,35,39,43] of the 8 studies with significant effects were conducted among undernourished and 3 [34,40,41] among healthy children.

**TABLE 3:** Characteristics of probiotic interventional studies

Author, publication year	Study country	Dietary intervention type, duration^1^	Method of bio-specimen analysis	Vehicle(s)	Sample size^2^ (age)	Health status	Growth outcome(s)	Gut microbiome outcome	Objective(s)	Main finding(s)	Significant positive effect?
1. Agustina et al., 2013 [40]	Indonesia	Four-arm study. 50 m/d low calcium milk or 440 mg/d regular calcium milk or regular calcium milk + 5 x 10^8^ CFU/d *Lactobacilluscasei* or regular calcium + 5 x 10^8^ CFU/d *L*. *reuteri* for 6 mo	Blood analysis	Low lactose milk	494 (1–5 y)	Healthy	Weight, height, WAZ, HAZ, hemoglobin, serum ferritin hematocrit	None	To investigate the hypotheses that cow milk with added probiotics would improve growth, iron, and zinc status of Indonesian children, whereas milk calcium alone would improve growth but reduce iron and zinc status.	*L. reuteri* modestly improved growth by increasing weight gain and weight and height velocity, whereas *L. casei* modestly improved weight velocity	Yes
2. Grenov et al., 2017 [33]	Uganda	Two-arm study. 5 x 10^9^ CFU/d *Bifidobacterium animalis* ssp*. lactis* + 5 x 10^9^ CFU/d *L. rhamnosus* or placebo maltodextrin for 8 to 12 wk or till recovery	Anthropometry only	F-75, F-100 or RUTF	314 (6–59 mo)	Undernourished	Weight	None	To assess the effect of probiotics on diarrhea or growth during in and out-patient treatment of children with SAM.	Results do not support using probiotics for the treatment of hospitalized children with SAM and severe medical complications	No
3. Hemalatha et al., 2014 [37]	India	Three-arm study. 2–5 x 10^9^ CFU/d *L*. *paracasei* or 2–5 x 10^9^ CFU/d *Bifidobacterium animalis* ssp. *lactis* or placebo for 9 mo	qPCR of stool	Milk	379 (2–5 y)	Healthy	Weight, height, WAZ, HAZ, WHZ	Bacterial count	To determine the effect of probiotics on diarrhea and growth in preschool children in a community setting.	Neither of the tested probiotics, *L. paracasei* or *B. lactis,* had any influence on weight gain or linear growth	No
4. Kamil et al., 2022 [35]	Indonesia	Two-arm study. 10^8–9^ CFU/d *L*. *plantarum or* placebo (Lactona) for 50 d	16S rRNA sequencing, qPCR, and gas chromatography of stool	Gummy skimmed milk powder	30 (37 ± 11.78 mo or 37 ± 12.98 mo)	Undernourished	Weight, height, WHZ, WAZ, HAZ	Alpha and beta-diversity	To evaluate the efficacy of gummy *L. plantarum* in preventing the progression of severe undernutrition.	*L. plantarum* has the potential to prevent the progression of severe undernutrition in infants	Yes
5. Kara et al., 2019 [39]	Turkey	Two-arm study. 10^9^ CFU/d *L. rhamnosus* or control for 3 mo	Blood analysis	Age-appropriate diet	71 (6 mo to 5 y	Undernourished	BMI, BMI *z*-score, albumin	None	To investigate the protective effects of *L. rhamnosus* in malnourished children in terms of incidence of infection, anthropometric and metabolic parameters.	Daily prophylactic use of *L. rhamnosus* in malnourished children prevents most infections and improves nutritional status	Yes
6. Kusumo et al., 2019 [38]	Indonesia	Four-arm study. 2.3 × 10^10^ CFU/d *L. plantarum* only or 2.3 × 10^10^ CFU/d *L. plantarum* + 20 mg/d zinc sulfate or 20 mg/d zinc only or control for 90 d	Blood analysis	maltodextrin	38 (12–24 mo)	Healthy	Weight, plasma transforming growth factor-β1	None	To investigate the probiotic function of *L*. *plantarum* in modulating immune response in young children.	The probiotic *L. plantarum* significantly increases the humoral immune response with no effect on growth	No
7. Mai et al., 2020 [36]	Vietnam	Two-arm study. 10^8^ CFU/mL/d *L*. *casei* or no probiotic for 12 wk	Anthropometry only	Fermented milk	1003 (3–5 y)	Nutrient deprived children (at-risk children)	Weight, height	None	To evaluate the efficacy of fermented milk containing *L*. *casei* on the incidence of constipation, diarrhea, and nutritional status.	Consumption of fermented milk containing *L. casei* prevented constipation and acute respiratory infection and may be useful for treating diarrhea and improving nutritional status	Yes
8. Rehman et al., 2020 [32]	Pakistan	Three-arm study. 3 x 10^9^ CFU/d *L*. *paracasei ssp. paracasei* or 6 x 10^9^ CFU/d *L*. *paracasei ssp. paracasei* or standard therapy (control) for 48 d	Hematologic analysis using venous blood	F-75, F-100 or RUTF	30 (6–59 mo)	Undernourished	Serum albumin, serum glutamate, serum glutamic pyruvate	None	To evaluate the effect of probiotic fortification on the 3 phases of SAM rehabilitation.	Probiotics supplementation markedly affected important blood parameters in SAM children. Both phase and dose exerted effects on parameters	Yes
9. Saran et al., 2002 [43]	India	Two-arm study. 5 x 10^9^ CFU/d *L*. *acidophilus* or the control group (isocaloric supplement) for 6 mo	Anthropometry only	Curd	100 (2–5 y)	Undernourished	Weight, height	None	To evaluate if regeneration of the damaged gut epithelium through the use of *Lactobacillus*-rich fermented foods may yield beneficial results	Six months of probiotic supplementation may be beneficial with respect to a decrease in diarrheal morbidity and accelerated growth	Yes
10. Silva et al., 2008 [34]	Brazil	Two-arm study. 10^8^ CFU/d *L. acidophilus* or control for 101 d	Anthropometry and blood analysis	Fermented milk beverage	109 (2–5 y)	Healthy (low–bioavailable-iron diet intake)	WAZ, WHZ, and HAZ, hemoglobin, serum ferritin, hematocrit, serum iron	None	To investigate the effect of iron fortification with probiotic bacteria in a milk beverage on the growth and iron status of preschool children.	The fortified beverage contributed to improved nutrient intake and nutritional status of the preschool children	Yes
11. Sur et al., 2011 [42]	India	Two-arm study. 6.5 x 10^9^ CFU/d *L. casei* or control drink for 12 wk	Multiplex PCR of stool	Nutrient drink (defatted milk)	3229 (1–5 y)	Healthy	WAZ	Gene-specific for enteric parasites	To examine the role of a probiotic in the prevention of acute diarrhea and its effects on growth.	Probiotic arm, compared to the nutrient arm, was not associated with any specific etiology. No effect on nutritional status	No
12. Surono et al., 2011 [41]	Indonesia	Two-arm study. 2.31 x 10^8^ CFU/d *Enterococcusfaecium* or placebo (maltodextrin) for 90 d	Blood and saliva analysis	Low-fat milk	79 (15–54 mo)	Healthy	Weight	None	To investigate the effect of *E. faecium* in milk on humoral immune response and on body weight of preschool children	Novel probiotic *E. faecium* had significant positive effects on immune response and on weight gain in preschool children	Yes

BMI, body mass index; CFU, cell forming unit; F-100, formula-100; F-75, formula-75; HAZ, height-for-age *z*-score; PCR, polymerase chain reaction; qPCR, qualitative polymerase chain reaction; rRNA, ribosomal ribonucleic acid; RUTF, ready-to-use therapeutic feed; SAM, severe acute malnutrition; WAZ, weight-for-age *z*-score; WHZ, weight-for-height *z*-score.

1 Mean (range) = 112.6d (minimum = 48d, maximum =270d).

2 Total sample size = 5876.

**FIGURE 3:**
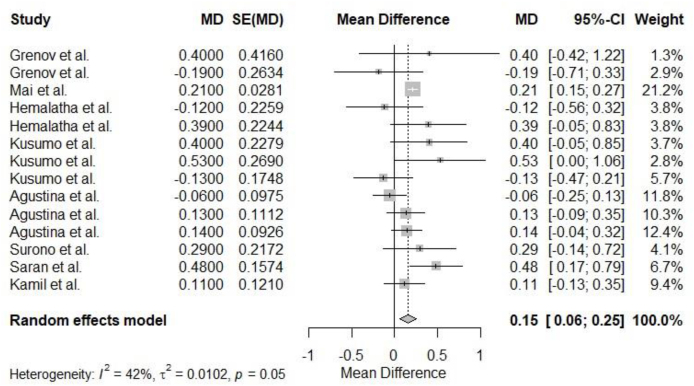
Forest plot of probiotic studies that reported weight gain. Growth (weight gain) was measured in kg. Weight gain was defined as the differences in preintervention weight and postintervention weight in the control group and the intervention group. MD referred to the arithmetic differences between the mean weight gain in the control group and the mean weight gain in the intervention group. CI, confidence interval; SE, standard error.

Figure 3 shows the forest plots with heterogeneity descriptions and the effect sizes MD of selected probiotic studies and growth outcome (weight gain) with 95% CI. Two of the included studies showed a positive effect on the intervention arm. Overall, there was no heterogeneity in the included studies (I^2^ = 42%, *P* = 0.05), and the cumulative effect size was statistically significant (MD = 0.15, 95% CI: 0.06, 0.25).

### Synbiotics

Table 4 contains the characteristics of study interventions that used synbiotics (a combination of prebiotics and probiotics) as a means of improving GM and growth. The studies analyzed a total of 2144 children. Seven studies met the inclusion criteria [[44], [45], [46], [47], [48], [49], [50]]. Four [44,46,48,50] of these studies were among children with undernutrition and 3 [45,47,49] among healthy children. The mean intervention duration was 154.4 d, with the least duration being 28 and the highest 365. Five [44,45,47,48,50] of the 7 studies (71.4%) reported a beneficial effect of synbiotics on ≥1 growth outcome in the intervention group as compared to the control group. Of the 5 synbiotic studies with beneficial effects, 3 [44,48,50] were among children with undernutrition and 2 [45,47] among healthy children. The vehicle(s) of administration were mainly F-100, RUTF, infant formula for children with undernutrition, and milk for healthy children, with 1 study using starch powder [48]. The specific synbiotics used in these studies include *B*. *infantis + Lacto-N-neotetraose* [44], GOS + *B*. *lactis* [45], long-chain FOS + short-chain GOS + *B*. *breve* [49]*, Bifidobacteria longum + L. rhamnosus + inulin +* FOS [47], Oligosaccharides + *B. lactis* [45,48] and *B. infantis* + Lacto-N-neotetraose [50].

**TABLE 4:** Characteristics of synbiotic interventional studies

Author, publication year	Study country	Dietary Intervention type, duration^1^	Method of bio-specimen analysis	Vehicle(s)	Sample size^2^ (age)	Health status	Growth outcome(s)	Gut microbiome outcome	Objective(s)	Main finding(s)	Significant positive effect?
1. Barratt et al., 2022 [50]	Bangladesh	Three-arm study. Probiotics 8 x 10^9^ CFU/d *Bifidobacterium infantis,* or 8 x 10^9^ CFU/d *B. infantis* + 1.6 g/d of Lacto-N-Neotetraose, or 625 mg/d lactose (placebo) for 28 d.	16S rRNA gene amplicon sequencing of stool	F-100 or infant formula	62 (2–6 mo)	Undernourished	WAZ, WHZ, MUAC.	Amplicon sequence variants	To examine how the treatment with probiotic, *B. infantis* strain with or without Lacto-N-Neotetraose supplementation, could colonize the gut microbiota of infants with SAM	Probiotic and synbiotic arms were associated with statistically significant improvements in ponderal growth (WAZ and MUAC)	Yes
2. Famouri et al., 2014 [48]	Iran	Two-arm study. Synbiotic (100 mg/d FOS + *Bacillus coagulans)* or control for 6 mo	Whole blood analysis	Starch powder	84 (12–55 mo)	Undernourished (failure to thrive)	Weight, height, head circumference	None	To assess the effect of synbiotics on growth indices of a sample of Iranian children with failure to thrive	This result has confirmed that the effect of synbiotics is significant in the weight gain of patients.	Yes
3. Firmansyah, et al., 2011 [47]	Indonesia	Two-arm study. Synbiotic (1 x 10^7^ CFU/d *Bifidobacteria longum +* 2 x 10^7^ CFU/d *Lactobacillus rhamnosus +* 1.02 g/d inulin *+* 2.38 g/d FOS) with milk or milk only for 12 mo	Fluorescence in-situ hybridization of stool	Cow milk-based formula	393 (12 mo)	Healthy	Weight, height, change in weight *z*-score	Bacterial counts	To evaluate the effects of milk containing synbiotics and lc PUFA on the growth of healthy 12-mo-old toddlers	Milk containing synbiotics and lc PUFA provide better growth and promote favorable gut colonization.	Yes
4. Kerac et al., 2009 [46]	Malawi	Two-arm study. RUTF only or RUTF with 10^11^ CFU/d of total probiotic (*Pediococcus pentosaceus,* + *Leuconostoc mesenteroides,* + *Lactobacillus paracasei* *ssp*. *paracasei and L*. *plantarum*) + 2.5 g/d of total prebiotics (oat bran + inulin + pectin + resistant starch) for 33 d (median) or till recovery	Anthropometry	F-100 and RUTF	795 (5–168 mo)	Undernourished	Weight, nutritional cure (WHZ >80% median)	None	To assess the effect of synbiotic functional food on improving existing treatments for SAM	Nutritional cure was similar in both synbiotic and control groups.	No
5. Kosuwon et al., 2018 [49]	Thailand	Two-arm study. Placebo or 5.4 g lc GOS + 0.6 g lc FOS + 1.1 x 10^10^ CFU/d *B*. *breve* for 12 wk	Fluorescence in-situ hybridization of stool	Young child formula	119 (1–3 y)	Healthy	Median weight increase, height, immunoglobulin A	SCFA content and microbiome composition (bacterial counts)	To determine the effect of consuming young child formula supplemented with sc GOS, lc FOS, and *B. breve* on a child’s development	There were no significant differences in height and weight between both groups at end-line	No
6. Nuzhat et al., 2023 [44]	Bangladesh	Three-arm study. Probiotic (8 x 10^9^ CFU/d *B. infantis*) or synbiotic (8 x 10^9^ CFU/d *B. infantis* +1.6 g/d Lacto-N-neotetraose) or placebo (lactose) for 4 wk	Only anthropometry	F-75, F-100, infant formula	67 (2–6 mo)	Undernourished	Change in rate of weight, HAZ	None	To explore the role of probiotic and synbiotic supplementation on the ponderal and linear growth of infants of 2–6 mo with SAM	Infants supplemented with *B. infantis* demonstrated better weight gain in comparison to the synbiotic or placebo	Yes
7. Sazawal et al., 2010 [45]	India	Two-arm study. Milk fortified with 2.4 g/d oligosaccharides + 1.9 x 10^7^ CFU/d *B*. *lactis* or control for 1 y	Hematologic analysis using venous blood	Milk powder	624 (1–4 y)	Healthy	Weight, Height, HAZ, WAZ, WHZ, hemoglobin, hematocrit, serum ferritin, plasma zinc	None	To evaluate the effect of *B*. *lactis* HN019 and prebiotic-fortified milk on iron status, anemia, and growth	Found a significant beneficial effect on weight velocity and lower risk of being iron deficient by consumption of synbiotic-fortified milk for 1 y.	Yes

CFU, cell forming unit; F-100, formula-100; FOS, fructo-oligosaccharides; GOS, galacto-oligosaccharides; HAZ, height-for-age *z*-score; lc FOS, long-chain fructo-oligosaccharides; lc GOS, long-chain galacto-oligosaccharides; lc PUFA, long-chain polyunsaturated fatty acid; MUAC, mid-upper arm circumference; PUFA, polyunsaturated fatty acid; rRNA, ribosomal ribonucleic acid; RUTF, ready-to-use therapeutic feed; SAM, severe acute malnutrition; sc GOS, short-chain galacto-oligosaccharides; SCFA, short-chain fatty acid; WAZ, weight-for-age *z*-score; WHZ, weight-for-height *z*-score.

1 Mean (range) = 153.4 d (minimum = 28 d, maximum =365 d).

2 Total sample size = 2144.

**FIGURE 4:**
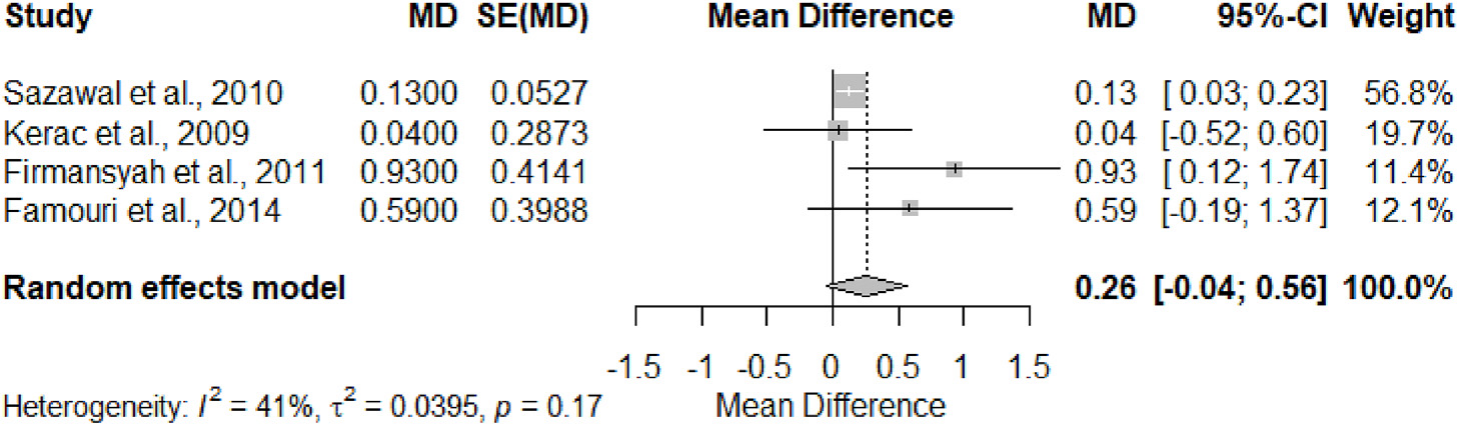
Forest plot of synbiotic studies that reported weight gain. Growth (weight gain) was measured in kg. Weight gain was defined as the differences in preintervention weight and postintervention weight in the control group and the intervention group. MD referred to the arithmetic differences between the mean weight gain in the control group and the mean weight gain in the intervention group. CI, confidence interval; SE, standard error.

Figure 4 shows forest plots with the computed MD and 95% CI for selected synbiotic studies. There is no significant heterogeneity (I^2^ = 41%, *P* = 0.17) among included studies. Although only 2 studies had significant effects on weight gain, the effect sizes are tilted toward the intervention arm. However, there are no overall significant differences in effect sizes (MD = 0.26, 95% CI: –0.04, 0.56) of synbiotic intervention on the growth outcome (weight gain).

### “Microbiome complementary feeds”

Table 5 shows the characteristics of “microbiome complementary feed” studies. Seven “microbiome complementary feeds” studies [[51], [52], [53], [54], [55], [56], [57]] met the inclusion criteria and were included for analysis. Out of these, 5 studies [[52], [53], [54],^56^,^57^] were conducted among healthy children, whereas 2 [51,55] were among children with undernutrition. The total number of children analyzed in these studies was 1635. The dietary interventions include Lipid-based Nutrient Supplement (LNS) [53], Microbiome-Directed complementary feed + Ready-to-use supplementary feed [51], fortified corn-soy blend + LNS [54], milk LNS + soya LNS + corn-soya blend [56], cowpea flour + corn-soya blend [57] and cowpea enriched F-75 / F-100 [55]. The mean intervention duration was 246.6 d, with the lowest and highest duration being 28 d and 540 d, respectively. In all, 2 [51,55] out of the 7 studies (28.6%) had significant effects on ≥1 growth outcome among the intervention group as compared to the control group. The 2 studies were among children with undernutrition. All included studies used the 16s rRNA sequencing except 1 study [54] that used a PCR method for microbial analysis.

**TABLE 5:** Characteristics of “microbiome complementary feed” studies directed at microbiome and growth

Author, publication year	Study Country	dietary intervention type, duration^1^	Method of bio-specimen analysis	Vehicle(s)	Sample size^2^ (age)	Health status	Growth outcome(s)	Gut microbiome outcome	Objective(s)	Main finding(s)	Significant positive effect?
1. Aakko et al., 2017 [54]	Malawi	Four-arm study. No intervention or 71 g/d micronutrient-fortified CSB or 54 g/d micronutrient-fortified LNS with milk protein base, or 54 g/d micronutrient-fortified LNS with soy protein base for 12 mo	Quantitative real-time PCR method of stool	N/A	160 (6–12 mo)	Healthy	Weight, stunting, HAZ	Bacterial counts	To assess the effect of LNS and CSB flour on *Bifidobacterium* and *Staphylococcus aureus* gut microbiota composition	The dietary supplementation did not have an effect on the *Bifidobacterium* and *Staphyloccusaureus* microbiota composition of the study among infants	No
2. Calder et al., 2021 [55]	Uganda	Three-arm study. Standard nutritional milk feeds with 35 g/L cowpea or 4.8 g/L inulin or placebo till resolved (maximum 28 d)	16S rRNA analysis of stool	F-75 or F-100	58 (7–59 mo)	Undernourished	Weight, proportion of weight gain (>5 g/kg/d)	SCFA counts, alpha-diversity	To investigate the use of a legume-enriched feed in the earliest stages of in-patient stabilization in acutely unwell children with SAM at the highest risk of death.	Legume-enriched feeds perform better than standard feeds in the treatment of SAM for mortality and weight gainFaecal bacterial richness and short-chain fatty acid concentration are preserved during antibiotic use	Yes
3. Chen et al., 2021 [51]	Bangladesh	Two-arm study. 50 g/d MDCF or 50 g/d RUSF (control) for 3 mo	Qualitative PCR and amplicons 16S rDNA of stool	N/A	118 (12–18 mo)	Undernourished	WHZ, WAZ, HAZ, MUAC	ASV abundance	To evaluate MDCF or RUSF on nutritional outcomes	Changes in the WHZ and WAZ are consistent with the benefit of MDCF on growth	Yes
4. Cheung et al., 2016 [56]	Malawi	Four-arm study. No supplementation (control) or 54 g/d milk LNS, 54 g/d soya LNS, or 71 g/d CSB for 12 mo	16S rRNA gene sequencing of stool	N/A	213 (6-mo-old children)	Healthy	Linear growth	Bacterial counts	To examine whether 2 forms of LNS or a Micronutrient-fortified CSB is associated with the development of the gut microbiota	Nutritional supplementation by LNS or CSB for 12 mo did not affect the gut microbiota profile	No
5. Hughes et al., 2020 [53]	Malawi	Two-arm study. 20 g/d LNS or non-LNS for 12 mo	16S rRNA gene sequencing of stool	N/A	512 (6–12 mo)	Healthy	Weight, WHZ, HAZ, WAZ, c-reactive protein	Shannon diversity and microbiome for age *z*-score	To determine whether the infant microbiota modified the effects of a randomized controlled trial of LNS on growth	Effects were not statistically significant, suggesting gut microbiota did not alter the effect of LNS on infant growth and inflammation	No
6. Ordiz et al., 2020 [57]	Malawi	Three-arm study. Cowpea flour or common beans flour or CSB flour (control) all arms at 80 kcal/d for 6–9 mo, 120 kcal/d for 9–12 mo for 24 wk	16S rRNA gene sequencing of stool	N/A	236 (6–12 mo)	Healthy	HAZ	Alpha-diversity, the relative abundance of ASV	To determine if a daily legume supplement given to Malawian infants aged 6–12 mo alters fecal microbiota and growth	Neither cowpea nor common bean altered the overall 16S configuration at any age. Cowpea supplementation improved linear growth from the ages of 6–9 mo	No
7. Robertson et al., 2023 [52]	Zimbabwe	Four-arm study. Standard of care or IYCF or WASH or IYCF + WASH for 18 mo. (no dosage)	Metagenome sequencing of stool	Complementary feed	335 (1–18 mo	Healthy	HAZ, WHZ, growth velocity	Alpha-diversity, beta-diversity	To map the assembly of the gut microbiome of children randomly assigned in the trial of improved water, sanitation, and hygiene and infant and young child feeding project	Early-life gut microbiome is unresponsive to the randomly assigned interventions intended to improve linear growth	No

ASV, amplicon sequence variant; CSB, corn-soya blend; F-100, formula-100; F-75, formula-75; HAZ, height-for-age *z*-score; IYFC, infant and young child feeding; LNS, lipid-based nutrient supplement; MDCF, microbiota-directed complementary food; MUAC, mid-upper arm circumference; PCR, polymerase chain reaction; rRNA, ribosomal ribonucleic acid; RUSF, ready-to-use supplementary food; SAM, severe acute malnutrition; SCFA, short-chain fatty acids; WASH, water sanitation and hygiene; WAZ, weight-for-age *z*-score; WHZ, weight-for height *z*-score; N/A; rDNA.

1 Mean (range) = 246.7 d (minimum = 28 d, maximum =540 d).

2 Total sample size = 1635.

**FIGURE 5:**
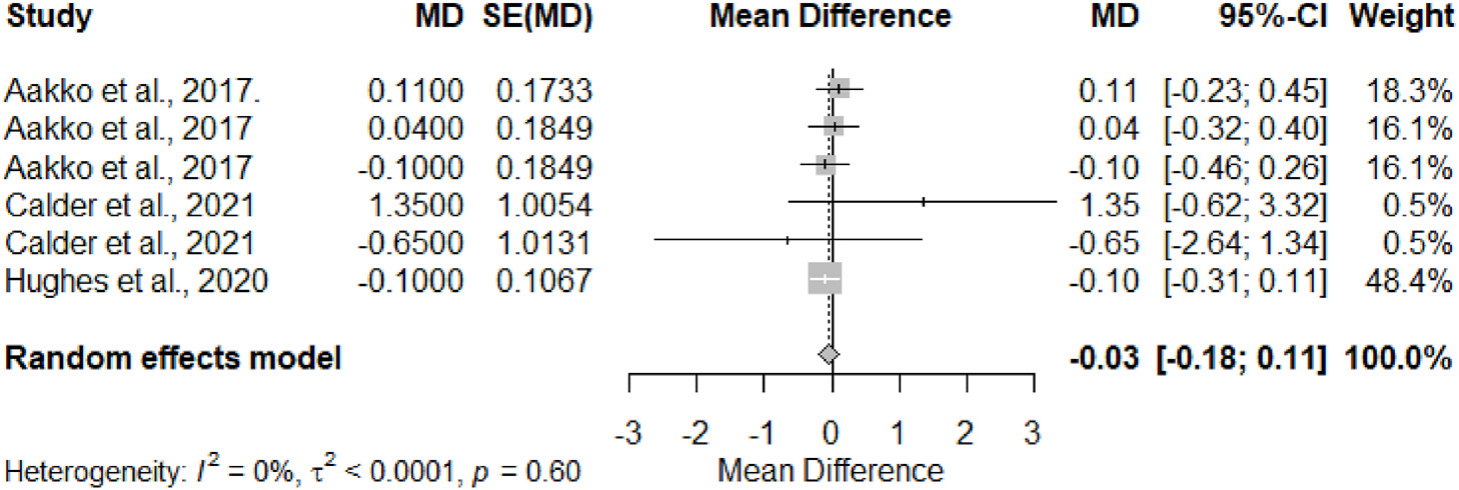
Forest plot “microbiome complementary feed” studies that reported weight gain. Growth (weight gain) was measured in kg. Weight gain was defined as the differences in preintervention weight and postintervention weight in the control group and the intervention group. MD referred to the arithmetic differences between the mean weight gain in the control group and the mean weight gain in the intervention group.

Figure 5 shows forest plots with the computed MD and 95% CI for selected “microbiome complementary feed” studies. There was no significant heterogeneity (I^2^ = 41%; *P* = 0.17) among included studies. One study [53] had a significant effect on weight gain, and generally, the effect sizes are tilted toward the intervention arm. However, no overall significant differences in effect sizes (MD = 0.26, 95% CI: –0.04, 0.56) of the “microbiome complementary feed” intervention on the outcome weight gain were detected.

### Growth as uniformly positive

This study classified all growth as better because a good number of studies were among children with malnutrition. In these instances, more growth may usually be good. Additionally, for studies among presumably “healthy” children, some interventions were conducted among participants who were at risk of undernutrition. For instance, 2 studies [36,54] described their participants as children “at risk” of undernutrition. The other studies described the location of their interventions as either rural [[25], [26], [27],^52^,^53^,^56^,^57^], low-resourced [40], urban slum [29,42], or shantytown [31]. A significant number of children living in these locations are more likely to be at risk or suffer from undernutrition. For these reasons, this study synthesized growth as uniformly positive and good throughout.

### Cochrane ROB tool

**FIGURE 6:**
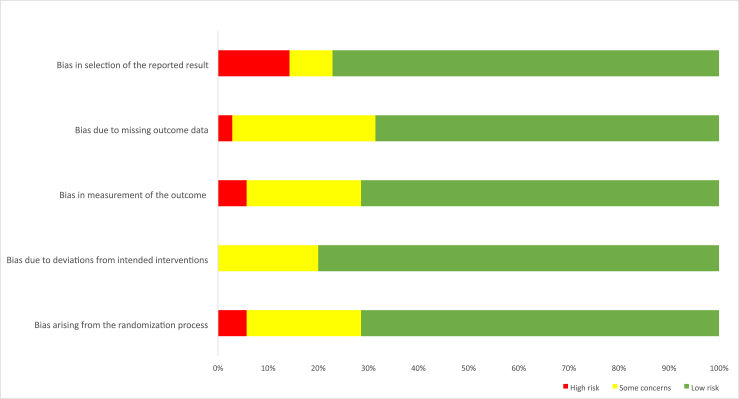
Evaluation of the risk of bias on growth outcome for all included studies.

Figure 6 shows ratings of the ROB tool 2.0 and the 5 domains for the individual control trials. This is a stacked bar plot, and the horizontal axis depicts the percentage risks in all included studies. Most of the studies were found to have a low ROB. The risks ROB assessments were conducted keeping in mind the tendency of funder influence. Moreover, as such, this study factored this into the rating of studies for ROB. Generally, studies, where funders either own the data or need to approve the manuscript, were termed as at risk of undue funder influence and were rated higher for ROB. It is also relevant to state that the majority of the studies were funded by industry, and all authors declared their conflicts of interest, indicating the funders did not have an influence on the outcome of the research, where necessary. Additional details on the individual scores of all included studies are attached as **Supplementary File 2.**

## Discussion

This systematic review aimed to synthesize existing evidence on dietary nutrition interventions targeting the child GM, assess the effectiveness of interventions on growth outcome, and identify research gaps and priorities for future studies. This present study involving 35 intervention studies with 11,047 children aged 0–5 y is 1 of the largest systematic reviews and meta-analyses on this subject in LMICs.

The qualitative results indicate that 5 out of 9 (55.6%) of prebiotic studies, 8 out of 12 (66.7%) of probiotic studies, 5 out of 7 (71.4%) of synbiotic studies, and 2 out of 7 (28.6%) of “microbiome complementary feed” studies had significant effects on ≥1 growth outcome in their respective intervention groups as compared with control groups. The effectiveness of synbiotics over other approaches at enhancing growth is consistent with the findings of another systematic review [8]. Synbiotics are a combination of probiotics and prebiotics, and this may have synergistic effects, where the probiotic microorganisms derive their food source from the prebiotic substrate, allowing them to grow and multiply more effectively [58]. The prebiotic substrate(s) is often specifically selected to stimulate the growth of the probiotic strain(s); this can result in more specificity and more targeted effects on the GM. Together, they provide a range of mutually beneficial effects on the GM, resulting in the reduction of inflammation [59], improving gut barrier function [60], and modulating the immune system [5], which could enhance growth. This kind of synergy may not be plausible in interventions that use only prebiotics or complementary feeds. Contrary to these findings, a systematic review involving 2971 infants with 25 control trials [15] could not establish that prebiotics or probiotics administered separately have lesser effects than when combined into a synbiotic. Their finding accentuates the need for probiotic strains to be well-matched to the specific prebiotic ingredient, or else their synergistic effects would not be harnessed.

This systematic review also revealed that prebiotics had a greater number of studies with significant effects among healthy children than children with undernutrition, whereas probiotics, synbiotics, and “microbiome complementary feeds” had more studies with significant effects among children with undernutrition than healthy children. For instance, in a probiotic study among children with undernutrition [32], *L. paracasei* had effects on growth outcomes, yet the same probiotic did not have effects among healthy children in another study [37]. These differences in effects are anticipated as the physiological states of healthy children and children with undernutrition may be different, as children in each group may respond differently even when exposed to similar nutritional interventions [3,61]. Healthy children may have an unperturbed GM, where prebiotics can act as a food source for the already existing beneficial bacteria, promoting their growth and activity. By enhancing the growth of these beneficial bacteria, prebiotics may help to improve overall health outcomes, including growth [62,63]. In children with undernutrition, however, GM may be perturbed and may have fewer beneficial bacteria. As such, introducing beneficial bacteria strains in the form of probiotics to restore diversity may be helpful in that context. Furthermore, the concurrent administration of probiotics and prebiotics may likely yield more beneficial outcomes. In sum, the findings of this study provide support for the proposition that the selection of a GM-targeted nutritional intervention aimed at enhancing child growth should be contingent upon the health status of the specific target population [8]. However, this should be interpreted with caution because of the very limited number of studies used in arriving at this finding. The finding should be recognized as preliminary and be a basis for future studies.

Generally, although GM-targeted nutrition interventions can have beneficial effects on growth, such effects may be more complex and depend on a variety of factors, including the specific probiotic strains or prebiotic substrates used and other characteristics such as breastfeeding, geographic location, antibiotics intake as well as individual host’s GM.

We expected that analysis of specimens of all nutrition-related GM studies would be conducted using fecal 16s rRNA sequencing. However, this review has revealed that only 4 [26,27,35,50] out of the 28 prebiotic, probiotic, and synbiotic studies used 16s rRNA sequencing. In addition, the low-cost blood hematologic analysis with anthropometric measurements, PCR [37,42,54], and fluorescence in-situ hybridization [47,49] are also acceptable approaches in resource-constrained situations where bacteria rRNA sequencing may not be feasible. Further, analysis of short-chain fatty acids, which include acetate, propionate, and butyrate, that have been associated with improved gut health and a more unperturbed GM [11] could also be used.

The role of infant feeding mode in the included studies can be categorized into 2 main contexts. The first context was mostly associated with breastmilk feeding. Because breastfeeding is associated with inducing higher proportions of *B. infantis*, a beneficial GM bacterium, supplementation provided to breastfeeding participants may offer no additional clinical benefits [31]. For instance, in some included studies that were conducted among breastmilk-fed infants [31,57], the lack of significant growth differences between the intervention and control groups was attributed to the beneficial effects of breastmilk feeding, reiterating the superiority of breastfeeding relative to other modes of infant feeding as a plausible explanation. However, this may require further inquiry as some studies [28,46,54], despite being among breastfed infants, concluded that insufficient dosage, effects of antibiotics, and dietary fiber intake are the reasons for the observed lack of significant differences. In other studies [28,29,40,47,49], the a priori knowledge of the beneficial effects of breastfeeding prompted authors to exclude breastfeeding infants from their studies. This was because of the challenges in measuring the exact quantities of human milk oligosaccharides and other bio-actives supplied through breastfeeding. This could confound with the dietary intervention and its effects on growth. In the second context, where the mode of feeding could limit intervention acceptability and adherence, some studies [25,26,31,39] used locally available complementary feeds as vehicles through which prebiotics and probiotics were administered. Additionally, in some studies [30,51] that used specialized feeds such as RUTF, F75, F100, or microbiome-directed complementary feed, mothers were asked to breastfeed before such therapeutic feeds were given or such feeds were given at half the daily recommended therapeutic dose so infants could still be breastfed.

### Limitations

There are several inherent limitations, as captured by included studies, that could help streamline and guide the direction of future studies. First, a number of studies [23,29,39,44], expressed concern about the influence of antibiotics intake and their inability to account for their effects on GM and growth. Some other studies [23,35,44,51,55] stated short intervention duration or short follow-up time as limitations in their interventions. The term short for these studies was an intervention duration of <3 mo and a follow-up time of <2 mo. As such, those studies could not measure long-term outcomes. Additional studies [25,31,35,40] stated that an underpowered sampled size calculation was a limitation. In those studies, although the sample size was powered enough to measure GM characteristics, it was not enough to detect differences in growth outcomes. Studies [25,35,49] expressed concern about not keeping dietary intake log books meant to measure other feeds. These studies were of the view that aside from the diet used for the intervention, there could be other foods that may have been consumed by participants, which may have gone unnoticed, but such feeds could exert influence on the GM and growth. As such, taking note of such foods would help explain certain unexpected outcomes or control for their confounding effects. Although some studies [26,27,36,39] expressed differences in baseline characteristics as a limitation, others [33,48] had reservations about the safety of probiotics in immune-suppressed children with undernutrition because of probiotic-induced sepsis. Finally, the lack of data on the volume of breastmilk consumed by infants [44] and challenges with sample collection [25], e.g., blood volume and storage process, were additional limitations stated by some authors. Hence, when planning sample collection procedures and storage, it is crucial to account for the half-lives of nutritional outcome measures and ensure adherence to appropriate sample collection procedures. The methodologic limitation of this review is also exogenous in that, because of differences in reported outcomes, not all articles were eligible to be included in the meta-analysis. The final limitation is the age range of included studies (0–5 y). Although meta-analyzing similar nutrition interventions over such a broad age range has been conducted in the past [16], the plausible confounding effects of the varying feeding modes during infancy and childhood in the context of this study should be acknowledged. This is particularly relevant as feeding mode differences, especially breastfeeding and complementary feeding, and their accompanying behavioral and developmental differences could have profound effects on GM and/or growth.

However, because all included interventions are studies with control groups, it is assumed that these limitations would be equally distributed among the intervention and the control groups such that their biased effects may not have significant differences on the outcome(s) of interest. It is presumed that such an assumption of random distribution may isolate only the intervention to be the sole cause of the observed differences between the arms at the end-line. These limitations notwithstanding, promising effects of some synbiotics, probiotic strains, prebiotic substrates, and some microbiome complementary feeds on growth among both undernourished and healthy children were detected in ∼60% of the included studies.

## Conclusion

Overall, 20 out of the 35 studies demonstrated significant effects on ≥1 growth outcome. For the qualitative analysis, the synbiotic studies had the highest number of studies significantly influencing growth, whereas the probiotic studies had significant effects on weight in the meta-analysis. However, the observed heterogeneity in prebiotic studies and lack of effectiveness in synbiotic and “microbiome complementary feed” groups could be because of the limited number of studies meta-analyzed. As a result, further intervention research is required to explore the effects of GM-targeted nutrition interventions on growth in LMICs. In future studies, antibiotic and breastmilk exposure should be accounted for. Accounting for antibiotics intake is important because it has the tendency to alter GM composition and possibly influence the effects of the interventions on growth. The sample size should be powered enough to measure not only the GM outcomes but also nutrition-related outcomes. Researchers interested in GM and growth intervention studies should aim for controlled trials with longer durations and follow-up periods of >3 and 2 mo, respectively. This would allow nutrition-related short-to-medium-term outcomes to be adequately assessed. Differences in baseline characteristics and sample collection variability resulting from collection and storage processes should also be well addressed.

In conclusion, the relative effectiveness of interventions was found to be dependent on the health status of participating children. Moreover, although probiotics and synbiotics may be effective at enhancing growth among children in LMICs, the selection of a microbiome-targeted nutrition intervention should be contingent upon the health status of the participating children.

## Author contributions

The authors’ responsibilities were as follows – HYA, AK: conceived this research; HYA, CA, AK: designed the research; HYA, CA: conducted research; HYA, CA: performed statistical analysis; HYA, CA, AK: wrote the paper; HYA, AK: had responsibility of final content; and all authors: read and approved the final manuscript.

### Conflict of interest

The authors report no conflicts of interest.

### Funding

The authors reported no funding received for this study.

### Data availability

This review’s protocol is registered with PROSPERO and available at www.crd.york.ac.uk/prospero/ as CRD42023434109.
