# Supplementary material for: Gut Microbiome-Targeted Nutrition Interventions and Growth among Children in Low- and Middle-Income Countries: A Systematic Review and Meta-Analysis
Source: Curr Dev Nutr. 2024 Feb 14;8(3):102085. doi: 10.1016/j.cdnut.2024.102085 (PMC10918490; doi:10.1016/j.cdnut.2024.102085)
Supplement: Multimedia component 1 [file mmc1.docx]

**SUPPLEMENTARY MATERIALS**

**SUPPLEMENTARY FILE II**

**Search strategy in identifying articles in PubMed, Cochrane Library & Google Scholar**

**PubMed**

Guided by MeSH vocabulary and the proposed topic’s objectives, the following search term was developed and used in PubMed.

**Supplementary Table 2: Search strategy in identifying prebiotic articles in PubMed**

| Number | Key words searched for articles on Prebiotics | # articles |
| --- | --- | --- |
| #1 | "Malnutrition"[Mesh] OR stunting[tiab] OR height[tiab] OR “linear growth“[tiab] OR wast*[tiab] OR growth[tiab] OR stunt*[tiab] OR HAZ[tiab] OR height-for-age[tiab] OR length-for-age[tiab] OR weight[tiab] OR weight-for-age[tiab] OR WAZ[tiab] OR weight-for-length[tiab] OR underweight[tiab] OR WHZ[tiab] OR WLZ[tiab] OR anaemia[tiab] OR “serum albumin”[tiab] | 3,079,096 |
| #2 | “1000 days”[tiab] OR "Child*"[Mesh] OR “toddler*” OR “under five” OR "Infant"[Mesh] OR child[tiab] OR infant*[tiab] OR “pre-school“[tiab] OR “pre school“[tiab] OR preschool[tiab] OR “ under-5”[tiab] OR “5 year*”[tiab] OR “five year*”[tiab] | 3,442,998 |
| #3 | "Dietary Supplements"[Mesh] OR prebiotic*[tiab] OR fructo-oligosaccharide*[tiab] OR fructooligosaccharide*[tiab] OR FOS[tiab] OR inulin[tiab] OR galacto-oligosaccharide*[tiab] OR galactooligosaccharide*[tiab] OR GOS[tiab] OR “rice bran”[tiab] OR “Infant formula”[tiab] OR milk[tiab] | 296,822 |
| #4 | #1 AND #2 AND #3 | 17,832 |
| #5 | "Clinical Trial" [Publication Type] OR "randomized controlled trial" OR "clinical trial" OR "RCT" OR "randomized control trial" OR "randomized clinical trial" | 1,159,938 |
| #6 | "Developing Countries"[Mesh] OR LMIC[tiab] OR “developing countr*”[tiab] OR “less developed countr*”[tiab] OR “less-developed countr*”[tiab] OR “Third-world countr*”[tiab] OR “under developed countr*”[tiab] or “under-developed countr*”[tiab] OR LMIC[tiab] OR “low and middle income countr*”[tiab] OR “low-to-middle-income countr*”[tiab] OR “low to middle income countr*”[tiab] OR “low-income”[tiab] OR “middle-income”[tiab] | 213,309 |
| #7 | #5 AND #6 | 8,641 |
| #8 | #4 AND #7 | 221 |

**Supplementary Table 3: Search strategy in identifying Probiotic articles in PubMed**

| Number | Key words searched for Probiotics | # articles |
| --- | --- | --- |
| #1 | "Malnutrition"[Mesh] OR stunting[tiab] OR height[tiab] OR “linear growth“[tiab] OR wast*[tiab] OR growth[tiab] OR stunt*[tiab] OR HAZ[tiab] OR height-for-age[tiab] OR length-for-age[tiab] OR weight[tiab] OR weight-for-age[tiab] OR WAZ[tiab] OR weight-for-length[tiab] OR underweight[tiab] OR WHZ[tiab] OR WLZ[tiab] OR anaemia[tiab] OR serum albumin[tiab] | 3,079,308 |
| #2 | “1000 days”[tiab] OR "Child*"[Mesh] OR “toddler*”[tiab] OR “under five”[tiab] OR "Infant"[Mesh] OR child[tiab] OR infant*[tiab] OR “pre-school“[tiab] OR “pre school“[tiab] OR preschool[tiab] OR “ under-5”[tiab] OR “5 year*”[tiab] OR “five year*”[tiab] | 3,443,077 |
| #3 | "Dietary Supplements"[Mesh] OR probiotic*[tiab] OR ferment*[tiab] OR bifidobacteri*[tiab] OR lactobaci*[tiab] OR Enteroco*[tiab] OR “Infant formula”[tiab] OR milk[tiab] | 427,677 |
| #4 | #1 AND #2 AND #3 | 21,406 |
| #5 | "Clinical Trial" [Publication Type] OR "randomized controlled trial" OR "clinical trial" OR "RCT" OR "randomized control trial" OR "randomized clinical trial" | 1,159,977 |
| #6 | "Developing Countries"[Mesh] OR LMIC[tiab] OR “developing countr*”[tiab] OR “less developed countr*”[tiab] OR “less-developed countr*”[tiab] OR “Third-world countr*”[tiab] OR “under developed countr*”[tiab] or “under-developed countr*”[tiab] OR LMIC[tiab] OR “low and middle income countr*”[tiab] OR “low-to-middle-income countr*”[tiab] OR “low to middle income countr*”[tiab] OR “low-income”[tiab] OR “middle-income”[tiab] | 213,319 |
| #7 | #5 AND #6 | 8,641 |
| #8 | #4 AND #7 | 226 |

**Supplementary Table 4: Search strategy in identifying Synbiotic articles in PubMed**

| Number | Key words searched for articles on synbiotics | #articles |
| --- | --- | --- |
| #1 | "Malnutrition"[Mesh] OR stunting[tiab] OR height[tiab] OR “linear growth“[tiab] OR wast*[tiab] OR growth[tiab] OR stunt*[tiab] OR HAZ[tiab] OR height-for-age[tiab] OR length-for-age[tiab] OR weight[tiab] OR weight-for-age[tiab] OR WAZ[tiab] OR weight-for-length[tiab] OR underweight[tiab] OR WHZ[tiab] OR WLZ[tiab] OR anaemia[tiab] OR serum albumin[tiab] | 3,079,308 |
| #2 | “1000 days”[tiab] OR "Child*"[Mesh] OR “toddler*”[tiab] OR “under five”[tiab] OR "Infant"[Mesh] OR child[tiab] OR infant*[tiab] OR “pre-school“[tiab] OR “pre school“[tiab] OR preschool[tiab] OR “ under-5”[tiab] OR “5 year*”[tiab] OR “five year*”[tiab] | 3,443,077 |
| #3 | "Dietary Supplements"[Mesh] OR (probiotic*[tiab] AND prebiotic*[tiab]) OR synbiotic*[tiab] OR probiotic*[tiab] OR ferment*[tiab] OR bifidobacteri*[tiab] OR lactobaci*[tiab] OR Enteroco*[tiab] OR fructo-oligosaccharide*[tiab] OR fructooligosaccharide*[tiab] OR FOS[tiab] OR inulin[tiab] OR galacto-oligosaccharide*[tiab] OR galactooligosaccharide*[tiab] OR GOS[tiab] OR “rice bran”[tiab] OR “Infant formula”[tiab] OR milk[tiab] | 473,108 |
| #4 | #1 AND #2 AND #3 | 21,636 |
| #5 | "Clinical Trial" [Publication Type] OR "randomized controlled trial" OR "clinical trial" OR "RCT" OR "randomized control trial" OR "randomized clinical trial" | 1,159,980 |
| #6 | "Developing Countries"[Mesh] OR LMIC[tiab] OR “developing countr*”[tiab] OR “less developed countr*”[tiab] OR “less-developed countr*”[tiab] OR “Third-world countr*”[tiab] OR “under developed countr*”[tiab] or “under-developed countr*”[tiab] OR LMIC[tiab] OR “low and middle income countr*”[tiab] OR “low-to-middle-income countr*”[tiab] OR “low to middle income countr*”[tiab] OR “low-income”[tiab] OR “middle-income”[tiab] | 213,319 |
| #7 | #5 AND #6 | 8,641 |
| #8 | #4 AND #7 | 227 |

**Supplementary Table 5: Search strategy in identifying complementary feed articles in PubMed**

| Number | Pubmed complementary feeds | # articles |
| --- | --- | --- |
| #1 | “Malnutrition”[Mesh] OR stunting[tiab] OR height[tiab] OR “linear growth“[tiab] OR wast*[tiab] OR growth[tiab] OR stunt*[tiab] OR HAZ[tiab] OR height-for-age[tiab] OR length-for-age[tiab] OR weight[tiab] OR weight-for-age[tiab] OR WAZ[tiab] OR weight-for-length[tiab] OR underweight[tiab] OR WHZ[tiab] OR WLZ[tiab] OR anaemia[tiab] OR serum albumin[tiab] | 3,079,308 |
| #2 | “1000 days”[tiab] OR “Child*”[Mesh] OR “toddler*”[tiab] OR “under five”[tiab] OR “Infant”[Mesh] OR child[tiab] OR infant*[tiab] OR “pre-school“[tiab] OR “pre school“[tiab] OR preschool[tiab] OR “ under-5”[tiab] OR “5 year*”[tiab] OR “five year*”[tiab] | 3,443,077 |
| #3 | "Dietary Supplements"[Mesh] OR “Gastrointestinal Microbiome”[tiab] OR Soybean*[tiab] OR vigna[tiab]  OR “Infant formula”[tiab] OR milk[tiab] | 289,486 |
| #4 | #1 AND #2 AND #3 | 17,879 |
| #5 | "Clinical Trial" [Publication Type] OR "randomized controlled trial" OR "clinical trial" OR "RCT" OR "randomized control trial" OR "randomized clinical trial" | 1,159,980 |
| #6 | "Developing Countries"[Mesh] OR LMIC[tiab] OR “developing countr*”[tiab] OR “less developed countr*”[tiab] OR “less-developed countr*”[tiab] OR “Third-world countr*”[tiab] OR “under developed countr*”[tiab] or “under-developed countr*”[tiab] OR LMIC[tiab] OR “low and middle income countr*”[tiab] OR “low-to-middle-income countr*”[tiab] OR “low to middle income countr*”[tiab] OR “low-income”[tiab] OR “middle-income”[tiab] | 213,319 |
| #7 | #5 AND #6 | 8,641 |
| #8 | #4 AND #7 | 218 |

**Cochrane Library**

**Supplementary Table 6: Search strategy in identifying Prebiotic articles in Cochrane Library**

| Number | Key words searched for prebiotic articles | # articles |
| --- | --- | --- |
| #1 | MeSH descriptor: [Malnutrition] explode all trees | 5,940 |
| #2 | (stunting OR height OR “linear growth“ OR wast* OR growth OR stunt* OR HAZ OR height-for-age OR length-for-age OR weight OR “weight-for-age” OR WAZ OR “weight-for-length” OR underweight OR WHZ OR WLZ OR anaemia OR “serum albumin”):ti,ab,kw | 216,238 |
| #3 | MeSH descriptor: [Child] explode all trees | 78,113 |
| #4 | (“1000 days” OR child* OR infant* OR pre-school OR "pre school" OR preschool OR under-5 OR "5 year" OR "five year"):ti,ab,kw | 241,427 |
| #5 | MeSH descriptor: [Prebiotics] explode all trees | 613 |
| #6 | ("Dietary Supplements" OR prebiotic* OR fructo-oligosaccharide* OR fructooligosaccharide* OR FOS OR inulin OR galacto-oligosaccharide* OR galactooligosaccharide* OR GOS OR “rice bran” OR “Infant formula” OR milk):ti,ab,kw | 31,561 |
| #7 | (#1 OR #2) AND (#3 OR #4) AND (#5 OR #6) | 5,610 |
| #8 | #7 in Trials | 5,466 |
| #9 | low income countr* OR middle income countr* OR LMIC OR low and middle income countr* OR low-to-middle-income countr* OR low to middle income countr* OR "low-income" OR "middle-income" OR Developing Countri* OR LMIC OR developing countr* OR less developed countr* OR less-developed countr* OR Third-world countr* OR under developed countr* OR under-developed countr* | 19,616 |
| #10 | MeSH descriptor: [Developing Countries] explode all trees | 1,143 |
| #11 | #9 OR #10 | 19,616 |
| #12 | #8 AND #11 | 421 |
| #13 | #12 in Trials | 272 |

**Supplementary Table 7: Search strategy in identifying Probiotic articles in Cochrane Library**

| Number | Key words searched for probiotic articles | # articles |
| --- | --- | --- |
| #1 | MeSH descriptor: [Malnutrition] explode all trees | 5,940 |
| #2 | (stunting OR height OR “linear growth“ OR wast* OR growth OR stunt* OR HAZ OR height-for-age OR length-for-age OR weight OR “weight-for-age” OR WAZ OR “weight-for-length” OR underweight OR WHZ OR WLZ OR anaemia OR “serum albumin”):ti,ab,kw | 216,238 |
| #3 | MeSH descriptor: [Child] explode all trees | 78,113 |
| #4 | (“1000 days” OR child* OR infant* OR pre-school OR "pre school" OR preschool OR under-5 OR "5 year" OR "five year"):ti,ab,kw | 241,427 |
| #5 | MeSH descriptor: [Probiotics] explode all trees | 3,058 |
| #6 | ("Dietary Supplements" OR probiotic* OR ferment* OR bifidobacteri* OR lactobaci* OR Enteroco* OR “Infant formula” OR milk):ti,ab,kw | 43,103 |
| #7 | (#1 OR #2) AND (#3 OR #4) AND (#5 OR #6) | 6,873 |
| #8 | #7 in Trial | 5,314 |
| #9 | MeSH descriptor: [Developing Countries] explode all trees | 1,143 |
| #10 | low income countr* OR middle income countr* OR LMIC OR low and middle income countr* OR low-to-middle-income countr* OR low to middle income countr* OR "low-income" OR "middle-income" OR Developing Countri* OR LMIC OR developing countr* OR less developed countr* OR less-developed countr* OR Third-world countr* OR under developed countr* OR under-developed countr* | 19,616 |
| #11 | #9 OR #10 | 19,616 |
| #12 | #8 AND #11 | 405 |
| #13 | #12 in Trials | 301 |

**Supplementary Table 8: Search strategy in identifying Synbiotic articles in Cochrane Library**

| **No.** | **Search Key Words for synbiotic articles** | **# articles** |
| --- | --- | --- |
| #1 | MeSH descriptor: [Malnutrition] explode all trees | 5,940 |
| #2 | (stunting OR height OR “linear growth“ OR wast* OR growth OR stunt* OR HAZ OR height-for-age OR length-for-age OR weight OR “weight-for-age” OR WAZ OR “weight-for-length” OR underweight OR WHZ OR WLZ OR anaemia OR “serum albumin”):ti,ab,kw | 216,238 |
| #3 | MeSH descriptor: [Child] explode all trees | 78,113 |
| #4 | (“1000 days” OR child* OR infant* OR pre-school OR "pre school" OR preschool OR under-5 OR "5 year" OR "five year"):ti,ab,kw | 241,427 |
| #5 | MeSH descriptor: [Synbiotics] explode all trees | 242 |
| #6 | ("Dietary Supplements" OR prebiotic* OR fructo-oligosaccharide* OR fructooligosaccharide* OR FOS OR inulin OR galacto-oligosaccharide* OR galactooligosaccharide* OR GOS OR “rice bran” OR “Infant formula” OR milk):ti,ab,kw | 31,561 |
| #7 | ("Dietary Supplements" OR probiotic* OR ferment* OR bifidobacteri* OR lactobaci* OR Enteroco* OR “Infant formula” OR milk):ti,ab,kw | 43,103 |
| #8 | (#1 OR #2) AND (#3 OR #4) AND (#5 OR #6 OR #7) | 6,939 |
| #9 | #8 in Trials | 6,767 |
| #10 | MeSH descriptor: [Developing Countries] explode all trees | 1,143 |
| #11 | low income countr* OR middle income countr* OR LMIC OR low and middle income countr* OR low-to-middle-income countr* OR low to middle income countr* OR "low-income" OR "middle-income" OR Developing Countri* OR LMIC OR developing countr* OR less developed countr* OR less-developed countr* OR Third-world countr* OR under developed countr* OR under-developed countr* | 19,616 |
| #12 | #10 OR #11 | 19,616 |
| #13 | #9 AND #12 | 470 |
| #14 | #13 in Trials | 361 |

**Supplementary Table 9: Search strategy in identifying complementary feeds articles in Cochrane Library**

| Number | Key words searched for probiotic articles | # articles |
| --- | --- | --- |
| #1 | MeSH descriptor: [Malnutrition] explode all trees | 5,940 |
| #2 | (stunting OR height OR “linear growth“ OR wast* OR growth OR stunt* OR HAZ OR height-for-age OR length-for-age OR weight OR “weight-for-age” OR WAZ OR “weight-for-length” OR underweight OR WHZ OR WLZ OR anaemia OR “serum albumin”):ti,ab,kw | 216,238 |
| #3 | MeSH descriptor: [Child] explode all trees | 78,113 |
| #4 | (“1000 days” OR child* OR infant* OR pre-school OR "pre school" OR preschool OR under-5 OR "5 year" OR "five year"):ti,ab,kw | 241,427 |
| #5 | MeSH descriptor: [Dietary Supplements] explode all trees | 16,970 |
| #6 | ("Dietary Supplements" OR “Gastrointestinal Microbiome” OR Soybean* OR vigna OR corn OR “Infant formula” OR milk):ti,ab,kw | 32,237 |
| #7 | (#1 OR #2) AND (#3 OR #4) AND (#5 OR #6) | 5,881 |
| #8 | #7 in Trial | 4,445 |
| #9 | MeSH descriptor: [Developing Countries] explode all trees | 1,143 |
| #10 | low income countr* OR middle income countr* OR LMIC OR low and middle income countr* OR low-to-middle-income countr* OR low to middle income countr* OR "low-income" OR "middle-income" OR Developing Countri* OR LMIC OR developing countr* OR less developed countr* OR less-developed countr* OR Third-world countr* OR under developed countr* OR under-developed countr* | 19,616 |
| #11 | #9 OR #10 | 19,616 |
| #12 | #8 AND #11 | 380 |
| #13 | #12 in Trials | 285 |

**Google Scholar Search Strategy**

The following phrases were used on the Google Scholar website

1. “*Nutritional intervention and gut microbiome*”
2. *“Prebiotic intervention and malnutrition”*
3. *“Probiotic intervention and malnutrition”*
4. *“Synbiotic intervention and malnutrition”*
5. *“Complementary feed interventions and gut microbiome”*

Additionally, words such as

*“child”*, *“infant”,* “*randomized control trial”*, “*pilot studies”, “trials”, “wasting”, “stunting”, “underweight”, “weight-for-height”, “weight-for-age”, “height-for-age”, “microbiome-for-age”, “α-diversity”, “β-diversity”, “anaemia”, “serum albumin”, “serum feritin”, “undernutrition”, “nutritional biomarkers”, “growth”, “Bifidobacteria”, “Lactobacilus”, “complementary feed”*

The above were used one after the other, as filters and limiters to provide specificity to retrieved articles.
