# Supplementary material for: Gut Microbiome-Targeted Nutrition Interventions and Growth among Children in Low- and Middle-Income Countries: A Systematic Review and Meta-Analysis
Source: Curr Dev Nutr. 2024 Feb 14;8(3):102085. doi: 10.1016/j.cdnut.2024.102085 (PMC10918490; doi:10.1016/j.cdnut.2024.102085)
Supplement: Multimedia component 2 [file mmc2.docx]

**SUPPLEMENTARY FILE III**

**Supplementary Table 10** Cochrane risk of bias evaluation for all included studies

| **Prebiotics studies** | | | | | |
| --- | --- | --- | --- | --- | --- |
| **Author & Year** | **(1) Bias arising from the randomization process;** | **(2) Bias due to deviations from intended interventions;** | **(3) Bias due to missing outcome data;** | **(4) Bias in measurement of the outcome;** | **(5) Bias in selection of the reported result;** |
| Batool et al., 2023 | Low risk | Low risk | Low risk | Low risk | Unclear |
| Rehman et al., 2020 | Low risk | Low risk | Unclear | Unclear | Low risk |
| Pfluger et al., 2022 | Low risk | Low risk | Low risk | Low risk | Low risk |
| Paganini et al. 2019 | Low risk | Low risk | Unclear | Unclear | Low risk |
| Zambrana et al., 2019 | Low risk | Low risk | Low risk | Unclear |  |
| Ribeiro et al. 2012 | Unclear | Low risk | Unclear | Low risk | High risk |
| Nakamura et al. 2006 | Low risk | Low risk | Low risk | Unclear | Low risk |
| Jones et al. 2015 | Low risk | Low risk | Unclear | High risk | Low risk |
| Duggan et al. 2003 | Low risk | Low risk | Unclear | Unclear | Low risk |
|  |  |  |  |  |  |
| **Probiotics Studies** | | | | | |
| **Author & Year** | **(1) Bias arising from the randomization process;** | **(2) Bias due to deviations from intended interventions;** | **(3) Bias due to missing outcome data;** | **(4) Bias in measurement of the outcome;** | **(5) Bias in selection of the reported result.** |
| Rehman et al., 2020 | Low risk | Low risk | Low risk | Low risk | Low risk |
| Grenov et al. 2017 | Low risk | Low risk | Low risk | Low risk | Low risk |
| Mai et al. 2020 | High risk | Unclear | Low risk | High risk | Low risk |
| Hemalatha et al. 2014 | Unclear | Low risk | Unclear | Low risk | Low risk |
| Kusumo et al. 2019 | Low risk | Low risk | Low risk | Unclear | Low risk |
| Kara et al. 2019 | Unclear | Unclear | Low risk | Low risk | High risk |
| Agustina et al. 2013 | Low risk | Low risk | Unclear | Low risk | Low risk |
| Surono et al. 2011 | Low risk | Unclear | Low risk | Low | Unclear |
| Sur et al. 2011 | Unclear | Low risk | Low risk | Low risk | High risk |
| Saran et al. 2002 | Low risk | Unclear | Low risk | Low risk | Low risk |
| Silva et al. 2008 | High risk | Low risk | Unclear | Unclear | Low risk |
| Kamil et al., 2022 | Low risk | Unclear | Low risk | Low risk | Low risk |
|  |  |  |  |  |  |
| **Synbiotics studies** | | | | | |
| **Author & Year** | **(1) Bias arising from the randomization process;** | **(2) Bias due to deviations from intended interventions;** | **(3) Bias due to missing outcome data;** | **(4) Bias in measurement of the outcome;** | **(5) Bias in selection of the reported result.** |
| Nuzhat et al., 2023 | Low risk | Low risk | Unclear | Low risk | Low risk |
| Sazawal et al. 2010 | Low risk | Low risk | Unclear | Low risk | Low risk |
| Kerac et al., 2009 | Low risk | Low risk | Low risk | Unclear | High risk |
| Firmansyah et al. 2011 | Unclear | Unclear | Low risk | Low risk | Unclear |
| Famouri et al. 2014 | Low risk | Low risk | High risk | Low risk | Low risk |
| Kosuwon et al. 2018 | Unclear | Low risk | Low risk | Low risk | Low risk |
| Barratt et al., 2022 | Unclear | Low risk | Low risk | Low risk | Low risk |
|  |  |  |  |  |  |
| **Complementary feeds study** | | | | | |
| **Author & Year** | **(1) Bias arising from the randomization process;** | **(2) Bias due to deviations from intended interventions;** | **(3) Bias due to missing outcome data;** | **(4) Bias in measurement of the outcome;** | **(5) Bias in selection of the reported result.** |
| Chen et al., 2021 | Low risk | Low risk | Low risk | Low risk | Low risk |
| Robertson et al., 2023 | Low risk | Low risk | Low risk | Low risk | Low risk |
| Hughes et al., 2020 | Low risk | Low risk | Low risk | Low risk | Low risk |
| Aakko et al., 2017 | Unclear | Low risk | Low risk | Low risk | Low risk |
| Calder et al., 2021 | Low risk | Low risk | Low risk | Low risk | Low risk |
| Cheung et al., 2016 | Low risk | Unclear | Low risk | Low risk | High risk |
| Ordiz et al., 2020 | Low risk | Low risk | Low risk | Low risk | Low risk |
